# Sympathetic nerve signals: orchestrators of mammary development and stem cell vitality

**DOI:** 10.1093/jmcb/mjae020

**Published:** 2024-05-13

**Authors:** Zi Ye, Yu Xu, Mengna Zhang, Cheguo Cai

**Affiliations:** Department of Pulmonary Oncology, Hubei Province Cancer Clinical Study Center, Hubei Key Laboratory of Tumor Biological Behaviors, Zhongnan Hospital of Wuhan University; Frontier Science Center for Immunology and Metabolism, Medical Research Institute, Wuhan University, Wuhan 430071, China; Department of Pulmonary Oncology, Hubei Province Cancer Clinical Study Center, Hubei Key Laboratory of Tumor Biological Behaviors, Zhongnan Hospital of Wuhan University; Frontier Science Center for Immunology and Metabolism, Medical Research Institute, Wuhan University, Wuhan 430071, China; Key Laboratory of Systems Health Science of Zhejiang Province, School of Life Science, Hangzhou Institute for Advanced Study, University of Chinese Academy of Sciences, Hangzhou 310024, China; Department of Pulmonary Oncology, Hubei Province Cancer Clinical Study Center, Hubei Key Laboratory of Tumor Biological Behaviors, Zhongnan Hospital of Wuhan University; Frontier Science Center for Immunology and Metabolism, Medical Research Institute, Wuhan University, Wuhan 430071, China; Key Laboratory of Systems Health Science of Zhejiang Province, School of Life Science, Hangzhou Institute for Advanced Study, University of Chinese Academy of Sciences, Hangzhou 310024, China

**Keywords:** mammary development, mammary stem cells, sympathetic nerve, mammary microenvironment

## Abstract

The mammary gland is a dynamic organ that undergoes significant changes at multiple stages of postnatal development. Although the roles of systemic hormones and microenvironmental cues in mammary homeostasis have been extensively studied, the influence of neural signals, particularly those from the sympathetic nervous system, remains poorly understood. Here, using a mouse mammary gland model, we delved into the regulatory role of sympathetic nervous signaling in the context of mammary stem cells and mammary development. Our findings revealed that depletion of sympathetic nerve signals results in defective mammary development during puberty, adulthood, and pregnancy, accompanied by a reduction in mammary stem cell numbers. Through *in vitro* three-dimensional culture and *in vivo* transplantation analyses, we demonstrated that the absence of sympathetic nerve signals hinders mammary stem cell self-renewal and regeneration, while activation of sympathetic nervous signaling promotes these capacities. Mechanistically, sympathetic nerve signals orchestrate mammary stem cell activity and mammary development through the extracellular signal-regulated kinase signaling pathway. Collectively, our study unveils the crucial roles of sympathetic nerve signals in sustaining mammary development and regulating mammary stem cell activity, offering a novel perspective on the involvement of the nervous system in modulating adult stem cell function and organ development.

## Introduction

The tissue microenvironment, often referred to as the adult stem cell niche, comprises not only neighboring cells but also extracellular matrix (ECM) components such as collagen, fibronectin, and laminin. Additionally, this microenvironment encompasses soluble factors secreted by and interacting with both cells and the ECM (Rauner and Kuperwasser, 2021). The intricate microenvironment, in conjunction with diverse cell lineages, orchestrates the precise organization of organs. The intricate crosstalk among these elements collectively gives rise to a harmonious organism that ensures normal organ development and physiological function (Polyak and Kalluri, 2010). Multiple tissues in adult mammals, including the skin (Gonzales and Fuchs, 2017), hair (Hu et al., 2021), blood (Comazzetto et al., 2021), liver (Mao et al., 2014), stomach (Sigal et al., 2017), and intestines (Barker, 2014), sustain an active regenerative state. This reliance on adult stem cells within these tissues facilitates tissue development, renewal, and regeneration, and these stem cell activities are under the regulation of the tissue microenvironment (Clevers et al., 2014). The pivotal contribution of adult stem cells to tissue development is widely acknowledged. For example, in the bone marrow and spleen, hematopoietic stem cells participate in adult bone marrow hematopoiesis, generating mature cells within the bone marrow to maintain adequate blood cell numbers and immune function (Comazzetto et al., 2021). Furthermore, during adipose tissue expansion, adipose stem cells undergo self-renewal and differentiation to maintain the structural integrity and function of adipose tissue (Quail and Dannenberg, 2019; Lecoutre et al., 2022). Despite extensive research on the role of the microenvironment in regulating stem cell behavior and tissue development, the precise functions and underlying molecular mechanisms of its components remain to be fully elucidated.

The mammary gland exhibits a bilayered epithelial structure, primarily composed of inner luminal (Lin^−^CD24^+^CD29^low^) cells and outer basal (Lin^−^CD24^+^CD29^hi^) cells (Shackleton et al., 2006). The proper development of the mammary gland relies on the correct self-renewal, differentiation, and formation of all mammary cell lineages by mammary stem cells (MaSCs) (Inman et al., 2015). MaSCs are located within the mammary basal layer (Prater et al., 2014), and they can reconstitute the entire mammary epithelial ductal structures in transplantation experiments (Stingl et al., 2006). Research has identified protein C receptor (Procr) as a marker for multipotent MaSCs. Procr-labeled MaSCs constitute 3%–8% of basal cells (Wang et al., 2015). MaSCs exist within a specialized microenvironment, often referred to as a niche, which comprises various cell types, including epithelial cells, immune cells, nerve cells, endothelial cells, adipocytes, and fibroblasts. The interactions and signal transduction between these cells collectively regulate the behavior of MaSCs (Wiseman and Werb, 2002; Need et al., 2014; Frank-Kamenetskii and Booth, 2019; Goff and Danforth, 2021). Investigating the intercellular crosstalk and signal transduction within this microenvironment is essential for comprehending the regulatory mechanisms governing MaSCs.

The mammary gland stands out as an organ with postnatal developmental characteristics (Inman et al., 2015). Moreover, it is a highly dynamic organ that undergoes significant structural and functional changes during puberty, pregnancy, lactation, and involution under the control of systemic hormones and environmental factors (Borellini and Oka, 1989; Visvader, 2009; Fu et al., 2020). At birth, only the mammary gland anlage is present, and the mammary epithelial cells originating from the nipple initially remain quiescent. During adolescence, hormonal regulation stimulates the significant elongation of mammary ducts into the mammary fat pad, leading to the formation of a highly branched ductal network, a process known as ductal morphogenesis (Lyons, 1958; Nandi, 1958). During gestation, driven by hormones such as progestin and prolactin, the mammary gland rapidly expands, giving rise to a network of alveolar cells capable of secreting milk during lactation. Subsequently, during involution, the expanded epithelium reverts to a resting state resembling that of adulthood through cell death and ECM remodeling (Van Keymeulen et al., 2011). In addition, the mammary gland morphology of adult female mice undergoes rapid changes during the cycling estrus cycle, including proestrus, estrus, metestrus, and diestrus (Nazario et al., 1994). Understanding the molecular mechanisms governing the periodic changes in mammary ducts is of great significance in the study of epithelial cell fate determination. The regulatory effects of various microenvironmental cells, such as stromal fibroblasts (Medina, 2004), macrophages (Kosenko et al., 2022; Zhou et al., 2023), and adipocytes (Couldrey et al., 2002; Landskroner-Eiger et al., 2010), on the morphology and structure of the mammary gland have been increasingly reported. However, limited knowledge exists regarding the regulatory influence of nerve cells and neural signals on mammary development.

The sympathetic nervous system (SNS) represents a crucial branch of the autonomic nervous system, emanating from the thoracolumbar spinal cord and innervating virtually all organ systems. The SNS mediates partial communication between the central nervous system and the periphery to maintain physiological homeostasis (Hanoun et al., 2015). In a steady state, the SNS maintains a baseline level of activity to oversee routine biological processes, including heart rate, respiration, and blood pressure, while also contributing to the ‘fight-or-flight’ response to acute stress (Borden et al., 2013; Karemaker, 2017). The SNS has been recognized as a significant component of several tissue microenvironments, including the skin (Shwartz et al., 2020), adipose tissue (Harlan et al., 2011; Wang et al., 2020), liver (Kiba, 2002), pancreatic islets (Borden et al., 2013), bone marrow (Katayama et al., 2006), and bone (Takeda et al., 2002). These findings underscore the connection between tissue homeostasis and the SNS, revealing that the SNS can modulate the activity of adult stem cells. Importantly, as a pivotal regulator of the tumor microenvironment, the SNS influences the progression and metastasis of breast cancer (Conceicao et al., 2021). Numerous studies have demonstrated that norepinephrine (NE) released from sympathetic nerves and adrenergic receptors are involved in regulating several hallmarks of breast cancer, including proliferation (Perez Pinero et al., 2012), survival (Sastry et al., 2007), angiogenesis (Chen et al., 2014; Zhou et al., 2020), immune evasion (Kamiya et al., 2019), ECM remodeling, and invasion (Chang et al., 2016; Pon et al., 2016). Nevertheless, it remains unclear whether the sympathetic nerves present in the mammary gland play a role in regulating mammary epithelial homeostasis and MaSC behavior.

In this study, we utilized a chemical model to investigate the physiological function of the SNS in regulating mammary development and MaSC activity by depleting the sympathetic nerves from mouse mammary glands. Additionally, through *in vitro* three-dimensional (3D) culture and *in vivo* transplantation analyses, we explored the relationship between MaSC activity and sympathetic nerve activity. Our investigation identifies the SNS as a critical regulatory factor within the mammary microenvironment, elucidates the molecular mechanisms underlying its regulation of mammary homeostasis, and provides a novel perspective for the study of SNS involvement in modulating adult stem cell function.

## Results

### 6-Hydroxydopamine administration depletes sympathetic nerves and delays mammary development

**Figure 1:**
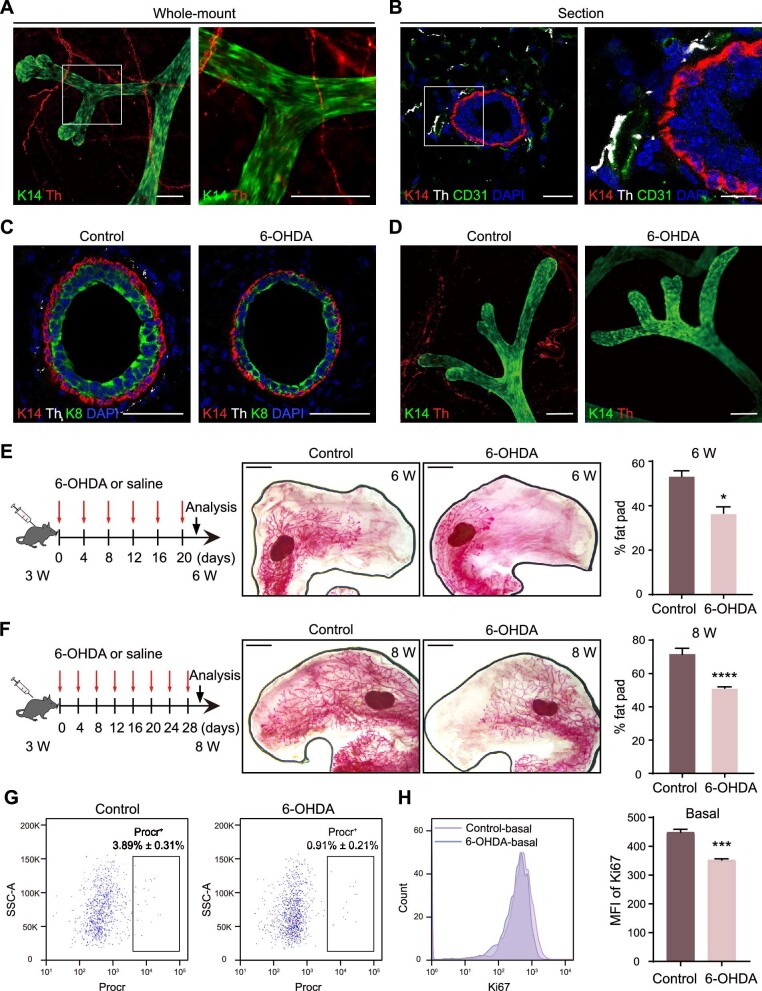
6-OHDA-induced sympathectomy delays mammary development and suppresses MaSC proliferation. (**A**) Whole-mount staining images depicting the spatial distribution of K14^+^ mammary basal cells alongside tyrosine hydroxylase-labeled (Th^+^) sympathetic nerve fibers within mouse mammary fat pad. Scale bar, 100 μm. (**B**) Immunofluorescence staining of K14, Th, and CD31 (vascular endothelial cell marker) in mouse mammary fat pad. Scale bar, 25 μm (left) or 10 μm (right). (**C**) Immunofluorescence staining of Th, K14, and K8 (luminal cell marker) in mouse mammary glands indicate fewer sympathetic nerve fibers in the 6-OHDA group. Control, control mice; 6-OHDA, 6-OHDA-treated mice. Scale bar, 50 μm. (**D**) Whole-mount staining images depicting the duct morphology and sympathetic nerves in mouse mammary glands. Scale bar, 100 μm. (**E**) Schematic representation of sympathetic nerve deletion in developing mammary glands from 3-week-old (3 W) to 6-week-old (6 W). Carmine staining of mammary glands and quantification of mammary duct elongation in 6 W pubertal female mice indicate the delay in mammary development from 3 W to 6 W. Scale bar, 2 mm. (**F**) Schematic representation of sympathetic nerve deletion in developing mammary glands from 3 W to 8 W. Carmine staining of mammary glands and quantification of mammary duct elongation in 8 W adult female mice indicate the blockade of mammary development from 3 W to 8 W. Scale bar, 2 mm. (**G**) FACS analysis of Lin^−^CD24^+^CD29^hi^, Procr^+^ MaSCs in mouse mammary glands. (**H**) FACS analysis (left) and quantification (right) of Ki67 expression in basal cells. MFI, mean fluorescence intensity. The results are representative of three independent experiments. Data are presented as mean ± SD. Student's *t*-test: *P < 0.05, ***P < 0.001, ****P < 0.0001.

We initiated the investigation by assessing the distribution of sympathetic nerve fibers within the mammary fat pad. Whole-mount staining analysis unveiled a complex interplay of the mammary ducts, sympathetic nerve fibers, and blood vessels that intricately adorned the mammary fat pad. Notably, the nerve fibers exhibited a spiral configuration closely associated with blood vessels (Figure 1A; Supplementary Figure S1A). Subsequently, immunofluorescence analysis revealed that sympathetic nerves were dispersed within the mammary fat pad, some close to the mammary ducts and some wrapping around the blood vessels (Figure 1B; Supplementary Figure S1B). Furthermore, our analyses extended to mammary tissues from various developmental stages, including the prepubertal phase (3 weeks of age), adolescence (5 weeks of age), and adulthood (8–12 weeks of age). Remarkably, the distribution of sympathetic nerves persisted throughout these developmental phases, underscoring their abundant presence within the mammary microenvironment (Supplementary Figure S1C and D).

To investigate the potential involvement of sympathetic nerves in the regulation of mammary development, we employed a subcutaneous injection of 6-hydroxydopamine (6-OHDA) in 3-week-old mice, a procedure known to induce transient damage to peripheral noradrenergic neurons without affecting the central nervous system (Livnat et al., 1985; Iversen et al., 1994), following established protocols (Kostrzewa and Jacobowitz, 1974). Immunofluorescence analysis provided compelling evidence of the efficacy of 6-OHDA treatment, as it significantly reduced the abundance of sympathetic nerve fibers in the mammary gland compared to the control group (Figure 1C; Supplementary Figure S1E and F), with nerve fiber density decreasing by ∼80% (Supplementary Figure S1G). Remarkably, this treatment did not affect mammary basal cells, luminal cells, and CD31^+^ vascular endothelial cells, as evidenced by microscopic analysis (Supplementary Figure S1E and F), and the absence of sympathetic nerves did not lead to overt alterations in mammary morphology (Figure 1D). Carmine staining analysis unveiled a noteworthy delay in mammary duct elongation following 3 or 5 weeks of 6-OHDA treatment (Figure 1E and F). Additionally, fluorescence-activated cell sorting (FACS) analysis revealed that 6-OHDA treatment did not cause significant changes in the major mammary epithelial cell populations, including basal cells, luminal cells, and mesenchymal cells (Supplementary Figure S1H), but strikingly reduced the proportion of Procr-labeled MaSCs (from 3.89% ± 0.31% to 0.91% ± 0.21%) (Figure 1G). Furthermore, FACS analyses demonstrated a significant decrease in Ki67^+^ basal or luminal cells in mice subjected to 6-OHDA treatment compared to the control group (Figure 1H; Supplementary Figure S1I). Collectively, these findings suggest that the SNS may play a pivotal role in the regulation of adolescent mammary development and MaSC proliferation.

### Neuroprotection mitigates mammary development impairment by 6-OHDA-induced sympathectomy 

**Figure 2:**
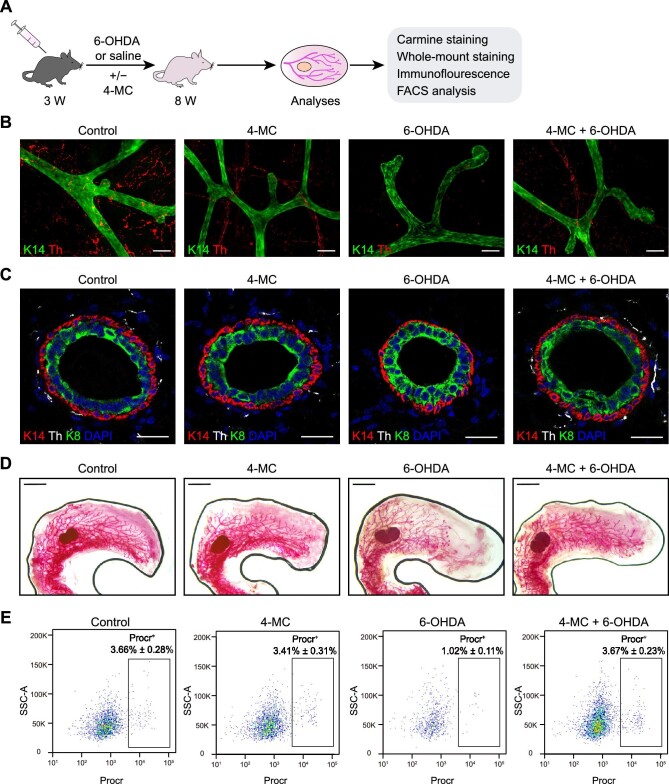
Neuroprotection reinstates normal mammary development. (**A**) Experimental design to assess whether 4-MC shields sympathetic fibers from 6-OHDA-induced sympathectomy. (**B**) Whole-mount staining of K14 and Th in mouse mammary glands. Control, saline-treated mice; 4-MC, 4-MC-treated mice; 6-OHDA, 6-OHDA-treated mice; 4-MC + 6-OHDA, 4-MC and 6-OHDA-treated mice. Scale bar, 100 μm. (**C**) Immunofluorescence staining of Th, K14, and K8 in mouse mammary glands. Scale bar, 25 μm. (**D**) Carmine staining of mammary glands. Scale bar, 2 mm. (**E**) FACS analysis of Procr^+^ basal cells. The results are representative of three independent experiments. Data are presented as mean ± SD.

The administration of 6-OHDA effectively eradicated sympathetic nerves and profoundly inhibited both mammary development and MaSC proliferations. To validate that the deleterious effects of 6-OHDA on mammary development stem from the absence of SNS, we introduced 4-methylcatechol (4-MC), which induces endogenous nerve growth factor production and shields sympathetic nerve fibers from clearance (Saita et al., 1996; Callizot et al., 2008), following established protocols (Figure 2A). Both whole-mount staining and immunofluorescence analyses unequivocally demonstrated that, compared to the 6-OHDA group, the nerve fibers in the 6-OHDA + 4-MC group remained largely intact, with minimal clearance (Figure 2B and C; Supplementary Figure S2A). This preservation of sympathetic nerve fibers did not cause substantial damage to the duct structure and morphology of the mammary epithelium (Figure 2B and C). Additionally, carmine staining demonstrated that 4-MC treatment effectively restored the normal elongation of mammary ducts that had been inhibited by 6-OHDA (Figure 2D; Supplementary Figure S2B). Furthermore, FACS analysis of mammary epithelial cells highlighted the ability of 4-MC treatment to reinstate the proportion of MaSCs (from 1.02% ± 0.11% to 3.67% ± 0.23%), which had been diminished by 6-OHDA, comparable to that of the control group (3.66% ± 0.28%) (Figure 2E). Collectively, these results affirm the indispensable role of sympathetic nervous signaling in orchestrating normal development of the mammary gland and the proliferation of MaSCs.

### Depletion of sympathetic nerves impairs mammary alveoli formation and adult homeostasis

**Figure 3:**
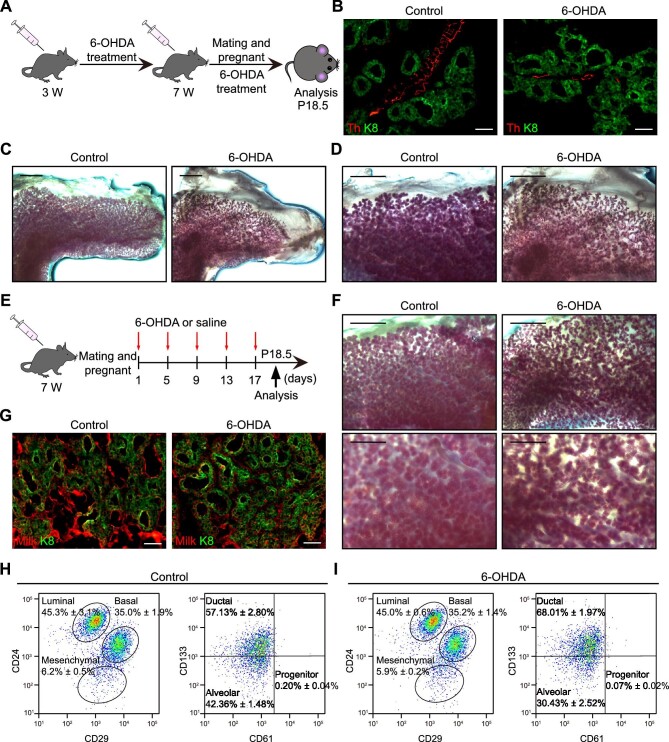
6-OHDA impairs mammary development during pregnancy. (**A**) A schematic representation of depleting sympathetic nerves from the prepubertal stage through pregnancy. (**B**) Immunofluorescence staining of Th and K8 in mammary glands. Scale bar, 50 μm. (**C**) Carmine staining depicting the extent of mammary duct elongation in pregnant mice. Scale bar, 2 mm. (**D**) Carmine staining illustrating the alveolar network in pregnant mice. Scale bar, 1 mm. (**E**) A schematic representation of depleting sympathetic nerves during pregnancy. (**F**) Carmine staining showcasing the alveolar network in pregnant mice. Scale bar, 2 mm (top) or 1 mm (bottom). (**G**) Immunofluorescence staining of milk and K8 in mammary glands. Scale bar, 50 μm. (**H** and **I**) FACS analysis of the subpopulations of luminal cells labeled by CD24, CD29, CD61, and CD133 in control (**H**) and sympathectomized (**I**) pregnant mice. The results are representative of three independent experiments. Data are presented as mean ± SD.

Pregnancy marks another period of profound mammary gland remodeling. To investigate whether the SNS plays a role in orchestrating the dynamic changes in the mammary gland during this pivotal stage, mice were subjected to 6-OHDA treatment commencing at 3 weeks of age, following established protocols (Figure 3A). The evaluation of mammary development was conducted on the 18.5th day of pregnancy (P18.5). As expected, 6-OHDA treatment led to a substantial reduction in sympathetic innervation within the mammary gland during pregnancy (Figure 3B; Supplementary Figure S3A), significantly reduced elongation of mammary epithelial ducts (Figure 3C; Supplementary Figure S3B), corroborating our earlier findings (Figure 1F), and impaired the formation of mammary lobular alveoli (Figure 3D; Supplementary Figure S3C). Notably, when mice were administered with 6-OHDA after pregnancy (Figure 3E), the formation of lobular alveoli was significantly compromised, resulting in a sparse alveolar network (Figure 3F), with the number of alveoli diminishing in a 6-OHDA-dose-dependent manner (Supplementary Figure S3D and E); however, the ability of alveolar cells to secrete milk was not lost (Figure 3G). Offspring evaluation showed that the pregnant mice in the 6-OHDA group generated fewer pups than those in the control group, but the weight of the pups was comparable to that of the control group, indicating that their lactation function was not affected (Supplementary Figure S3F and G). Furthermore, FACS analysis unveiled that 6-OHDA treatment led to a significant reduction in the proportion of luminal progenitor cells (from 0.20% ± 0.04% to 0.07% ± 0.02%) and alveolar cells (from 42.36% ± 1.48% to 30.43% ± 2.52%) (Figure 3H and I). Cumulatively, these data underscore the involvement of sympathetic nerves in the regulation of mammary remodeling during pregnancy.

In addition to puberty and pregnancy, the mammary duct morphology undergoes rapid dynamic changes during the estrus cycle in adulthood under the fluctuations of estrogen and progesterone (Sternlicht et al., 2006). To explore whether the sympathetic nerve is also crucial for adult mammary homeostasis, 6-OHDA was administered to adult mice to remove sympathetic nerves, and mammary glands were collected from mice at different estrus stages, including proestrus, estrus, metestrus, and diestrus, which were identified by vaginal cytology (Byers et al., 2012; Supplementary Figure S3H). Carmine staining demonstrated that the 6-OHDA group had significantly fewer side branches at diestrus (Supplementary Figure S3I and J), and FACS analysis confirmed that the proportion of Procr^+^ MaSCs significantly decreased in the 6-OHDA group at all stages (Supplementary Figure S3K). Collectively, these data suggest that the SNS plays an essential role in maintaining mammary homeostasis during the estrus cycle.

### Sympathetic nerve injury attenuates MaSC activity

**Figure 4:**
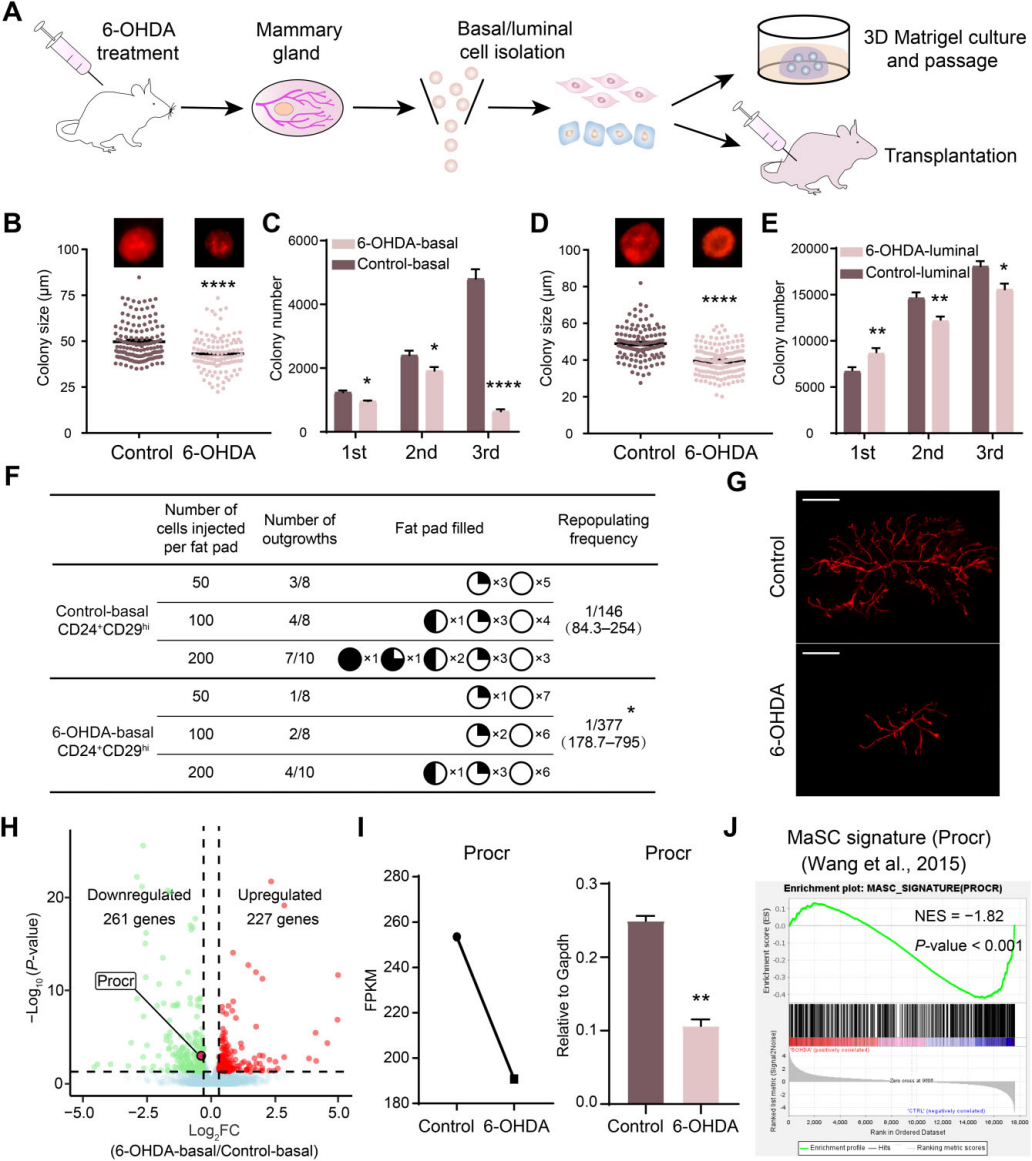
Sympathetic nerve injury impairs MaSC activity. (**A**) A schematic representation of *in vitro* 3D Matrigel culture and *in vivo* transplantation experiments. (**B** and **C**) The size (**B**) and number in serial passages (**C**) of colonies derived from basal cells in 3D culture. (**D** and **E**) The size (**D**) and number in serial passages (**E**) of colonies derived from luminal cells in 3D culture. (**F**) Mammary gland reconstitution of mammary basal cells in transplantation. (**G**) Whole-mount images illustrating typical mammary outgrowths after transplantation of basal cells. Scale bar, 2 mm. (**H**) A volcano plot depicting the differentially expressed genes in basal cells (log_2_FC > 0.3 and adjusted *P*-value <0.05). FC, fold change. (**I**) FPKM value and relative expression level of *Procr* in basal cells. FPKM, fragments per kilobase million. (**J**) GSEA demonstrating the downregulation of the gene signature ‘MaSC signature (Procr)’ in 6-OHDA-treated basal cells. The results are representative of three independent experiments. Data are presented as mean ± SD. Student's *t*-test: *P < 0.05, **P < 0.01, ****P < 0.0001.

The next pivotal question is whether sympathetic nerve injury hampers MaSC activity. To unravel this, we isolated mammary basal cells enriched with MaSCs and luminal cells from DsRed mouse subjected to 6-OHDA treatment, followed by *in vitro* 3D culture and *in vivo* transplantation analyses (Figure 4A). The 3D Matrigel culture revealed that colonies derived from basal cells of mice treated with 6-OHDA exhibited significantly smaller size compared to those from control mice (Figure 4B). In serial passages, the number of colonies formed by basal cells from mice treated with 6-OHDA markedly diminished compared to the control group (Figure 4C). Similarly, both the size and number in serial passages of colonies formed by luminal cells from mice treated with 6-OHDA were significantly reduced compared to those from control mice (Figure 4D and E). *In vivo* transplantation analysis revealed that the repopulating frequency of mammary basal cells subjected to 6-OHDA treatment was significantly reduced compared to the control group (from 1/146 to 1/377) (Figure 4F). Furthermore, whole-mount images demonstrated that control basal cells were capable of regenerating a more complete mammary gland, whereas basal cells treated with 6-OHDA only regenerated small-sized mammary ducts (Figure 4G). These findings collectively suggest that sympathetic nerve injury by 6-OHDA diminishes the activity of MaSCs.

To gain a deeper understanding of the regulatory role of the SNS in regulating MaSC activity, we conducted transcriptomic analysis of basal cells using RNA sequencing (RNA-seq). Volcano plot analysis unveiled significant downregulation of 261 genes and upregulation of 227 genes following 6-OHDA treatment to deplete sympathetic nerves (Figure 4H). Remarkably, the gene representative of active MaSCs, such as *Procr*, was enriched among the downregulated genes, and the decreased expression level of *Procr* in basal cells after 6-OHDA treatment was further verified by RNA-seq and real-time quantitative polymerase chain reaction (RT-qPCR) (Figure 4I). Furthermore, gene set enrichment analysis (GSEA) indicated that the gene signature of Procr-labeled MaSCs (Wang et al., 2015) was negatively enriched in 6-OHDA-treated basal cells (Figure 4J). In summation, these findings establish the critical role of sympathetic nerve signals in sustaining MaSC activity.

### Sympathetic nerves regulate mammary development and MaSC activity through NE–adrenergic receptors

**Figure 5:**
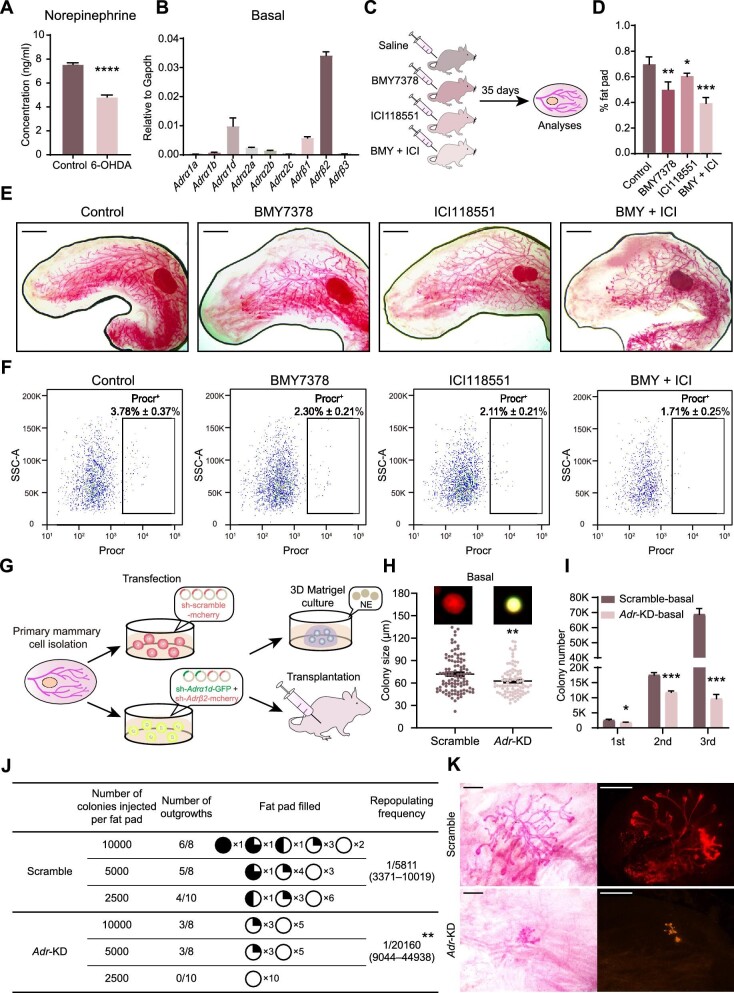
Sympathetic nerve signals maintain mammary development and MaSC activity via NE–adrenergic receptors. (**A**) Quantification of NE content in the mammary gland of control and sympathectomized mice. (**B**) RT-qPCR analysis of relative expression levels of adrenergic receptors in basal cells. (**C**) A schematic representation of pharmacologically blocking adrenergic receptors. (**D**) Quantification of mammary duct elongation in mice. (**E**) Carmine staining of mouse mammary glands. Scale bar, 2 mm. (**F**) FACS analysis of Procr^+^ basal cells in mouse mammary glands. (**G**) Experimental setup for knocking down *Adrα1d* and *Adrβ2* in primary mammary cells, followed by *in vitro* 3D Matrigel culture and *in vivo* transplantation. (**H**) Colony size of basal cells cultured in Matrigel. (**I**) Number of basal cell colonies in serial passages. (**J**) Mammary gland regeneration of primary mammary cells in transplantation. (**K**) Whole-mount images of typical mammary outgrowths after transplantation of primary mammary cells. Scale bar, 1 mm (left) or 2 mm (right). The results are representative of three independent experiments. Data are presented as mean ± SD. Student's *t*-test: *P < 0.05, **P < 0.01, ***P < 0.001, ****P < 0.0001.

Next, we sought to determine the specific receptors and intracellular signaling pathways mediating the regulation of mammary development and MaSC activity by the SNS. Sympathetic nerve terminals release NE, a neurotransmitter that binds to adrenergic receptors on target cells to exert function (Elenkov et al., 2000; Cole et al., 2015; de Lucia et al., 2019). The sympathectomy-induced reduction in NE levels in the mammary fat pad (Figure 5A) strongly suggested the sympathetic nerve as a primary source of NE in the mammary gland. RT-qPCR analyses revealed that the β2 adrenergic receptor (*Adrβ2*) and α1d adrenergic receptor (*Adrα1d*) were the dominant adrenergic receptors expressed in basal cells, luminal cells, mesenchymal cells, and Procr^+^ basal cells (Figure 5B; Supplementary Figure S4A). Subsequently, we administered mice with selective antagonists against ADRα1d (BMY7378) and ADRβ2 (ICI118551), followed by analyses after 35 days (Figure 5C). Carmine staining analysis indicated that treatment with a single antagonist (BMY7378 or ICI118551) modestly inhibited ductal elongation, while simultaneous treatment with both antagonists (BMY + ICI) resulted in a significant inhibition of mammary duct elongation, akin to the effect observed in 6-OHDA-treated mice (Figure 5D and E). Notably, whole-mount staining and immunofluorescence analyses revealed that antagonist treatment did not alter the morphology and structure of mammary ducts (Supplementary Figure S4B and C). Furthermore, FACS analysis indicated that, compared to the control group (3.78% ± 0.37%), the cell population of Procr-labeled MaSCs in the BMY + ICI group (1.71% ± 0.25%) was significantly reduced (Figure 5F). Moreover, the amount of Ki67^+^ basal cells significantly decreased after antagonist treatment (Supplementary Figure S4D), suggesting the reduction in basal cell division and proliferation.

To gain deeper insights into the role of adrenergic receptors, we used lentiviral vectors to knock down these receptors in primary mammary cells, followed by *in vitro* 3D culture and *in vivo* transplantation analyses to assess MaSC activity (Figure 5G). The effectiveness of *Adrβ2* and *Adrα1d* knockdown was confirmed by RT-qPCR analysis (Supplementary Figure S4E). *In vitro*, simultaneous knockdown of *Adrα1d* and *Adrβ2* (*Adr*-KD) significantly reduced the size and number in serial passages of basal cell colonies (Figure 5H and I) and luminal cell colonies (Supplementary Figure S4F). *In vivo*, individual knockdown of *Adrα1d* or *Adrβ2* did not affect the number of mammary outgrowths but reduced their size (Supplementary Figure S4G and H), while simultaneous knockdown of *Adrα1d* and *Adrβ2* significantly reduced the repopulating frequency of mammary basal cells (from 1/5811 to 1/20160) (Figure 5J and K). These results collectively demonstrate that ADRα1d and ADRβ2 mediate the regulatory effects of the SNS on mammary development and MaSC activity.

### Activation of adrenergic signaling enhances MaSC activity

**Figure 6:**
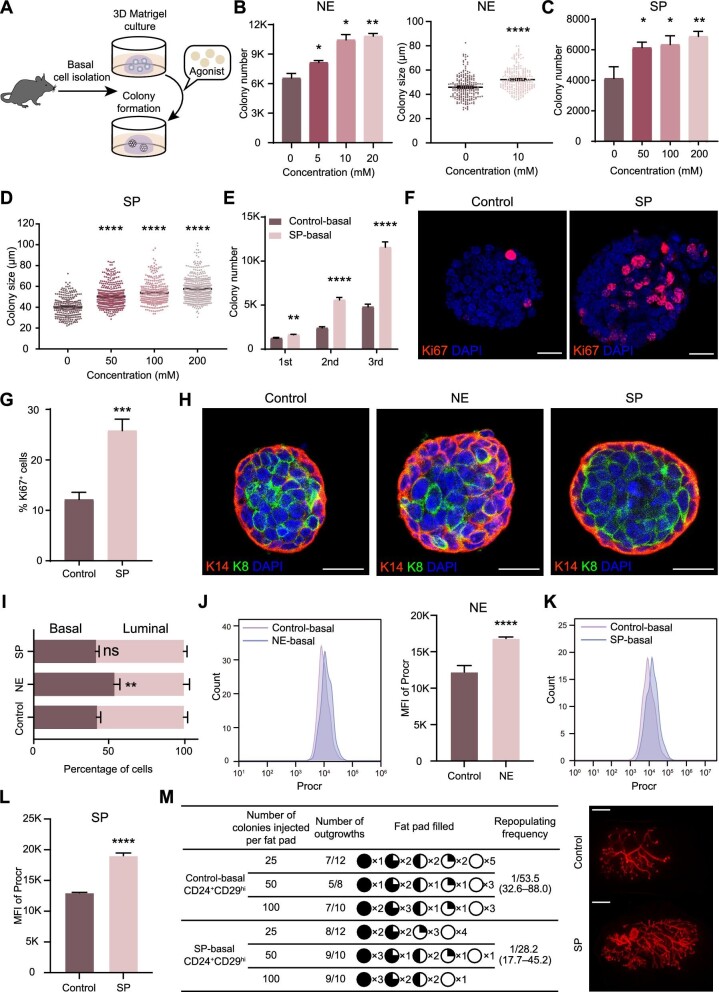
Activation of adrenergic signaling enhances MaSC self-renewal. (**A**) A schematic representation of activating adrenergic receptors during *in vitro* Matrigel culture. (**B**) The number (left) and size (right) of basal cell colonies following NE treatment in Matrigel culture. (**C** and **D**) The number (**C**) and size (**D**) of basal cell colonies after SP treatment in Matrigel culture. (**E**) The number of basal cell colonies in serial passages. (**F** and **G**) Representative immunofluorescence images (**F**) and quantification (**G**) of proliferative cells (Ki67-labeled) in basal cell colonies. Scale bar, 20 μm. (**H** and **I**) Representative immunofluorescence images (**H**) and quantification (**I**) of basal cells (K14^+^) and luminal cells (K8^+^) in basal cell colonies. Scale bar, 20 μm. (**J**–**L**) FACS analysis and quantification of Procr expression in colonies formed by control, NE-treated (**J**), and SP-treated (**K** and **L**) basal cells. (**M**) Mammary gland regeneration of basal cell colonies in transplantation (left). Whole-mount images of typical mammary outgrowths after transplantation of basal cell colonies (right). Scale bar, 2 mm. The results are representative of three independent experiments. Data are presented as mean ± SD. Student's *t*-test: ns, not significant; *P < 0.05, **P < 0.01, ***P < 0.001, ****P < 0.0001.

To investigate whether activating the NE–adrenergic receptor signaling axis could enhance MaSC activity, we isolated mammary basal cells and cultured them in Matrigel in the presence of nonselective NE, the natural neurotransmitter of the SNS, or a combination of the selective β2 adrenoceptor agonist salbutamol and the selective α1 adrenoceptor agonist phenylephrine (SP) (Figure 6A). Colony formation analysis revealed that both the size and number of basal cell colonies increased with NE (Figure 6B) and SP in a dose-dependent manner (Figure 6C and D). In the presence of SP, the number of basal cell colonies significantly increased in serial passages (Figure 6E). Notably, luminal cell colony formation was also dose-dependently enhanced by NE or SP (Supplementary Figure S5A–D). Moreover, the percentage of Ki67^+^ cells in SP-treated basal cell colonies was significantly higher than that in control colonies (Figure 6F and G). To further investigate the effect of adrenergic signaling on cell fate, we performed K14 (basal cell marker) and K8 (luminal cell marker) staining on basal cell-derived colonies. The addition of NE increased the proportion of basal cells (from 41.93% ± 2.84% to 53.47% ± 4.09%) and decreased the proportion of luminal cells (from 58.06% ± 2.83% to 46.51% ± 4.73%), while SP treatment did not significantly change the proportion of basal and luminal cells (Figure 6H and I). FACS analysis of cultured basal cell colonies indicated that both NE and SP treatments significantly enhanced the proportion of Procr^+^ basal cells (MaSCs) (Figure 6J–L). When transplanted into the cleared fat pads of immunocompromised recipients, SP-treated colonies exhibited higher repopulating frequency compared to control colonies (from 1/53.5 to 1/28.2) (Figure 6M).

Furthermore, we conducted RNA-seq and transcriptomic analyses on cultured basal cell colonies. Volcano plot analysis revealed 414 significantly upregulated genes and 399 significantly downregulated genes in SP-treated colonies (Supplementary  Figure S5E). Gene Ontology (GO) enrichment analysis indicated that the genes significantly upregulated in SP-treated colonies were enriched in biological processes associated with epithelial cell proliferation, gland development, regeneration, developmental growth, morphogenesis, and nervous system process (Supplementary  Figure S5F). Additionally, GSEA indicated that gene signatures ‘mammary stem cell’, ‘mammary regeneration’, and ‘MaSC signature (Procr)’ were all significantly enriched in SP-treated colonies (Supplementary  Figure S5G). These findings collectively demonstrate that ADRβ2- and ADRα1d-mediated adrenergic signaling plays a crucial role in enhancing MaSC activity.

### Extracellular signal-regulated kinase signaling mediates the regulation of mammary development and MaSC activity by sympathetic nerve signals

**Figure 7:**
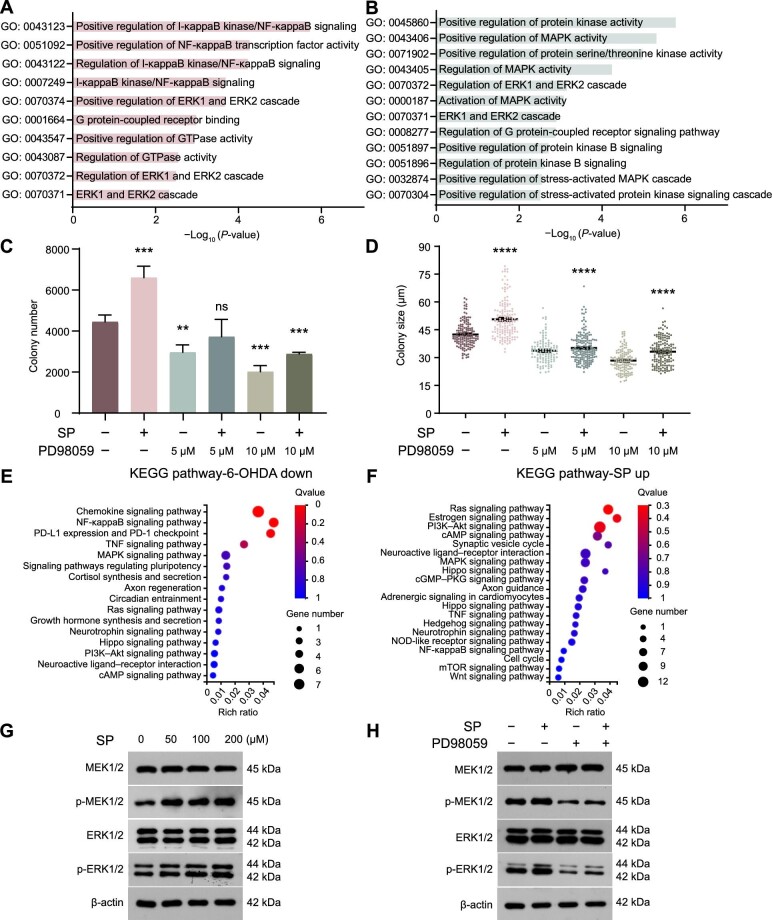
Sympathetic nerve signals activate the ERK pathway. (**A**) GO enrichment analysis of the downregulated genes in basal cells upon 6-OHDA treatment. (**B**) GO enrichment analysis of the upregulated genes in basal cell colonies stimulated by SP. (**C** and **D**) Colony number (**C**) and size (**D**) of basal cells treated with SP and/or PD98059 in Matrigel culture. (**E**) KEGG pathway analysis of the downregulated genes in 6-OHDA-treated basal cells. (**F**) KEGG pathway analysis of the upregulated genes in colonies formed by SP-treated basal cells. (**G** and **H**) Western blot analysis depicting the expression and phosphorylation of key ERK signaling molecules in primary mammary cells subjected to SP and/or PD98059 treatment. The results are representative of three independent experiments. Data are presented as mean ± SD. Student's *t*-test: ns, not significant; **P < 0.01, ***P < 0.001, ****P < 0.0001.

Subsequently, we conducted RNA-seq and transcriptomic analyses on basal cells treated with 6-OHDA and basal cell colonies treated with SP. GO enrichment analysis of differentially expressed genes revealed that the downregulated genes in 6-OHDA-treated basal cells and the upregulated genes in SP-treated basal cell colonies were associated with extracellular signal-regulated kinase (ERK) cascade and mitogen-activated protein kinase (MAPK) activity (Figure 7A and B). We then examined whether ERK signaling mediates the regulatory effects of sympathetic nerve signals on MaSC activity. Inhibiting ERK signaling with the ERK inhibitor PD98059 significantly hindered basal cell colony formation, leading to a dose-dependent reduction in both the number and size of the colonies, and also nullified the enhancing effect of SP on basal cell colony formation (Figure 7C and D). Furthermore, Kyoto Encyclopedia of Genes and Genomes (KEGG) analysis revealed that the genes associated with the MAPK signaling pathway (ERK signaling is one of the four classical MAPK pathways) were upregulated by SP but downregulated by 6-OHDA (Figure 7E and F). Interestingly, cell cycle-related pathway was enriched with SP-upregulated genes (Figure 7F), aligning with our earlier observation that SP enhanced the proliferation of basal cell colonies (Figure 6F and G). Western blot analysis confirmed that SP treatment significantly activated ERK signaling, as evidenced by the phosphorylation of ERK and mitogen-activated protein kinase kinase (MEK) (Figure 7G). Importantly, the inhibitor PD98059 effectively eliminated this ERK signaling activation (Figure 7H). These findings collectively indicate that ERK signaling acts as the intermediary in the regulation of mammary development and MaSC activity by sympathetic nerve signals.

## Discussion

In our study, we explored the pivotal role of the SNS in the regulation of mammary development and MaSC activity. Our findings reveal that sympathetic nerves, which are abundant in the mammary gland, play a crucial role in shaping mammary homeostasis by orchestrating intricate signaling pathways, most notably the ERK pathway.

The maintenance of mammary gland homeostasis and MaSC activity is the result of complex interactions between systemic hormones and microenvironmental cues (Asselin-Labat et al., 2010; Polyak and Kalluri, 2010; Wagers, 2012). While extensive research has uncovered the ECM and various cell types, including fibroblasts (Avagliano et al., 2020), immune cells (Rahat et al., 2016), and adipocytes (Couldrey et al., 2002; Landskroner-Eiger et al., 2010), within the mammary microenvironment, which not only provide a scaffold for the mammary gland but also regulate the fate of mammary epithelial cells through paracrine, physical, and hormonal interactions (Radisky, 2012), the understanding of the role of neural innervation in mammary homeostasis is still limited. Our study provides a critical insight by demonstrating that sympathetic nerves are a prominent component of the mammary microenvironment, exerting a profound influence on organ development and MaSC activity.

Moreover, the role of SNS in breast cancer has been noted in various studies. By α and β adrenergic receptors, the SNS can impact various molecular processes involved in tumor progression, including DNA damage repair, inflammation, angiogenesis, and tumor cell migration and invasion (Cole et al., 2015). In light of our findings, which highlight the significant impact of the SNS on mammary development and MaSC activity, it is reasonable to posit that regulating neural signals could offer a novel approach to influencing mammary development and potentially preventing the onset of breast cancer and other mammary diseases.

Our results also contribute to the growing body of knowledge regarding the influence of sympathetic nerves on adult stem cells (Katayama et al., 2006; Heidt et al., 2014; Zeng et al., 2019). While hair follicle stem cells respond to both basal and modestly elevated sympathetic tones, such as in cold environments, through synaptic-like connections (Shwartz et al., 2020), melanocyte stem cells only respond when sympathetic nerves are hyperactivated under conditions of severe stress (Zhang et al., 2020). Our study now adds MaSCs to the list of adult stem cells directly regulated by sympathetic innervation, although the precise cellular mechanisms underlying this regulation remain to be fully elucidated.

The MAPK/ERK pathway plays an important role in controlling various cellular processes, including cell survival, cell fate transition, growth, migration, proliferation, and death (Guo et al., 2020; Wen et al., 2022). Numerous reports have illustrated the contribution of ERK signaling to mammary epithelial cell proliferation (Cao et al., 2021), mammary epithelial tube elongation (Huebner et al., 2016), and proper mammary development (Larive et al., 2012). Furthermore, ERK signals mediate the MaSC stemness maintenance and self-renewal (Liu et al., 2022). Our study reveals that sympathetic nerve signals serve as a novel upstream activator of ERK, thus contributing to the regulation of MaSC activity and mammary development.

In conclusion, our research unveils the intricate functional and molecular mechanisms through which the SNS regulates MaSC activity and mammary development. These insights not only broaden our understanding of mammary regeneration but also offer a fresh perspective on the etiology of breast diseases, potentially paving the way for novel strategies for improving mammary health and combating breast-related conditions.

## Materials and methods

### Experimental animals

C57BL/6 and Balb/c-nude mice were purchased from Nanjing Biomedical Research Institute of Nanjing University. Actin-DsRed mice were obtained from Jackson Laboratories. All animals were maintained in a specific-pathogen-free animal facility in the Animal Center of Medical Research Institute of Wuhan University. The experimental procedures were approved by the Animal Care and Use Committee of Wuhan University.

### Cell culture and transfection

The HEK293T cell line was obtained from Shanghai Cell Bank Type Culture Collection Committee or American Type Culture Collection. For cell culture, HEK293T and primary mammary cells were cultured in Dulbecco's modified Eagle medium (DMEM) (high glucose, Hyclone) supplemented with 10% fetal bovine serum (FBS; PAN Biotech), 1% penicillin/streptomycin (Hyclone), and 0.3 mg/ml L-glutamine with or without the ERK inhibitor PD98059 (MCE), ADRβ2 agonist salbutamol (MCE), and ADRα1d agonist phenylephrine (MCE) at 37°C in a 5% CO_2_ incubator.

Plasmids were prepared using Endofree plasmid kit (Tiangen Biotech). Lentivirus was packaged by transient transfection in HEK293T cells. Briefly, HEK293T cells were co-transfected with vesicular stomatitis virus G, packaging plasmid Delta 8.9, and transfer vector, using the PEI (Sigma). At 48 h post-transfection, the culture medium was harvested and centrifuged at 3220× *g* for 5 min. Virus particles were stored at −80°C. Following viral infection, GFP- or mCherry-positive cells were sorted by FACS.

### 6-OHDA treatment

To induce acute peripheral sympathectomy during puberty, mice (3 weeks old) received subcutaneous injections of 6-OHDA (Sigma, 150 mg/kg). To induce sympathetic nerve damage during pregnancy, pregnant mice received subcutaneous injections of 6-OHDA (50 or 100 mg/kg). Two days after the last injection of 6-OHDA, the mice were euthanized for analyses.

### Inhibition of adrenergic receptors

To inhibit α1d and β2 adrenergic signaling in C57BL/6 mice (3 weeks old), the ADRα1d-specific antagonist BMY7378 (Selleck, 2 mg/kg) or ADRβ2-specific antagonist ICI118551 hydrochloride (Selleck, 1 mg/kg) was intraperitoneally injected daily for 5 weeks. For simultaneous ADRα1d and ADRβ2 pharmacological blockade, BMY7378 and ICI118551 were injected 3 h apart to minimize drug interactions.

### Neuroprotection induced by 4-MC

To protect the sympathetic nerve from 6-OHDA injury, mice (3 weeks old) received daily intraperitoneal injections of 4-MC (Sigma, 10 μg/kg) for 5 weeks starting from the same day as the first injection of 6-OHDA.

### Primary mammary single cell preparation

Mammary fat pads were isolated from female mice at the specified age. The fresh mammary tissue was finely minced, transferred into the appropriate volume of digestion medium, i.e. RPMI 1640 (Hyclone) with 5% FBS, 1% penicillin/streptomycin, 25 mM HEPES (Sigma), and 300 U/ml collagenase 3 (Worthington), and then digested for 2 h at 37°C, with a good shaking every 15 min. The digested tissue was centrifuged at 4°C, 805× *g*, for 5 min, and Red Blood Cell Lysis Buffer (Sigma) was added for 5 min incubation at room temperature. Single cell suspension was obtained by incubation with 0.05% trypsin–ethylenediaminetetraacetic acid (EDTA) (Hyclone) for 5 min at 37°C and 0.1 mg/ml DNase I (Sigma) for another 5 min at 37°C, followed by filtration through 70-μm cell strainers (Corning) and resuspension.

### FACS analysis and cell sorting

For mammary epithelial cell analysis, the following antibodies were used in 1:200 dilution to label cells: fluorescein isothiocyanate-conjugated CD31, CD45, TER119 (BD Biosciences), CD24-PE/cy7, CD29-APC, CD61-PE, CD133-BV421 (Biolegend), Procr-PE (eBioscience), biotinylated CD31, CD45, TER119 (BD Biosciences), and streptavidin-APC-eFluor780 (eBioscience). Antibody incubation was performed on ice for 20–25 min in phosphate-buffered saline (PBS) with 5% FBS, avoiding light. For Ki67 staining, after the mammary epithelial staining, the cells were fixed with 4% paraformaldehyde (PFA) for 30 min and then stained with Ki67-PE antibody (eBioscience, 1:200 dilution) for 20–25 min. 4,6-Diamidino-2-phenylindole (DAPI, Invitrogen) was added to distinguish viable cells. Cells were filtered through 50-μm cell strainers for sorting or analysis. Single cell sorting was performed using Beckman Moflo Astrois EQ. FACS analysis was performed using Beckman cytoflex or LSRFortessa X20. FACS data were analyzed using FlowJo_V10 software.

### RNA isolation, RNA-seq, and RT-qPCR

Primary cells or cultured colonies were lysed in RNAiso plus (TaKaRa). Total RNA was extracted following the manufacturer's instructions. Total RNA concentration was determined with Nanodrop2000. RNA-seq libraries were prepared according to the manufacturer's instructions, followed by sequencing on HiSeq X-10 sequencer (MGISEQ2000). The complementary DNA was generated from reverse-transcribed RNA using the PrimeScript RT Master Mix (TaKaRa). RT-qPCR was performed by a CFX system (Bio-Rad) with a FastStart Universal SYBR Green Master Mix Kit (Roche). Relative mRNA level was normalized to the *Gapdh* mRNA level. The mixture reacted under the following conditions: 10 min at 95°C, followed by 40 cycles of 15 sec at 95°C and 1 min at 60°C.

### Whole-mount staining

Mammary glands harvested from female mice were cut into 3 mm × 3 mm and then placed in digestion medium for 40 min at 37°C. Dissected mammary glands were fixed in 4% PFA for 1 h at 4°C. Fixed tissues were washed three times with PBST (PBS with 0.1% Triton X-100) for 5 min and then blocked by 1 ml maleate buffer containing Tween-20 (with 10% FBS) for 1 h at room temperature. The tissues were incubated with primary antibodies in blocking buffer overnight at 4°C, followed by washes, and then incubated overnight at 4°C with secondary antibodies. After washing, the tissues were put into 3 ml 80% sucrose overnight at 4°C with a good shaking. The next day, tissues were mounted with ProLong Gold Antifade Reagent (Cell Signaling Technology) with DAPI for 8–12 h before imaging. Images were taken by an optical microscope (ZEISS).

### Immunofluorescence

Mammary glands collected from female mice were cut into small pieces and fixed for 2 h in 4% PFA at 4°C. Fixed tissues were washed three times with PBS for 5 min and then transferred to a 30% sucrose solution overnight at 4°C. Samples were embedded in O.C.T. Compound (Tissue Tek), sectioned in a cryostat (12 μm thick) using the CryotomeFSE (Thermo Fisher), and mounted on CFSA 4× slides. Frozen sections were hydrated with PBST for 15 min at room temperature. Next, the sections were blocked in blocking buffer, PBS with 10% goat serum (Gibco^TM^) and 0.1% Triton X-100, for 2 h at room temperature. After blocking, the sections were incubated with primary antibodies at 4°C overnight, washed with PBST for three times, 5 min per time, and then incubated with the secondary antibodies for 1 h at room temperature, followed by washes. Finally, the sections were mounted with ProLong Gold Antifade Reagent with DAPI.

For staining colonies, Matrigel was dissolved by dispase (BD Biosciences) for 15 min at 37°C. Then, colonies were collected and fixed with 4% PFA for 30 min, followed by washes, blocking, and incubation with antibodies. Images were captured by a laser confocal scanning microscope (ZEISS).

### Carmine staining

The fourth pair of mammary glands collected from female mice were fixed in 4% PFA at 4°C for 2 h, washed with PBS for three times, 5 min per time, and incubated with carmine alum dye liquor overnight. The stained tissues were decolorized in the solution (50% ethanol with 2% HCl) for 2 h, with the solution refreshed every half hour, followed by dehydrating in 75%, 85%, 95%, 100%, and 100% ethanol for 1 h. Then, the tissues were permeabilized with Histo-Clear for 30 min. Images were captured by a stereoscopic microscope (Leica).

### Colony formation and passage assay

Sorted cells by flow cytometry were resuspended at a density of 1 × 10^6^ cells/ml in chilled 100% Matrigel (Thermo Fisher) with homogeneous mixing, clustered 30 ml/well of 48 wells, and plated in a 37°C incubator for polymerization. The mixture was cultured in DMEM/F12 (Hyclone) with 50 ng/ml epidermal growth factor (BD Biosciences) and insulin–transferrin–selenium (Sigma Aldrich, 1:100) with or without the ERK inhibitor PD98059 (MCE), NE, salbutamol, and phenylephrine. The medium was changed every 2 days. Analyses were performed after 7–9 days of culture.

For colony passage, Matrigel was dissolved by dispase for 15 min at 37°C. Then, the colonies were released from Matrigel and incubated in 0.25% trypsin–EDTA for 5 min at 37°C to obtain single cells, which were resuspended in Matrigel as described above.

### Mammary fat pad transplantation

Basal cell colonies or sorted cells were resuspended in 50% Matrigel with 25% FBS, 25% PBS, and 0.04% Trypan Blue (Sigma). The mixture was injected in a volume of 10 μl into the cleared fat pad of 3-week-old female Balb/c-nude mice. Reconstituted mammary outgrowths were harvested and analyzed 45–60 days after transplantation. Images were captured by a stereoscopic microscope (Leica).

### Mouse estrus cycle identification

Virgin female C57BL/6 mice aged 8–12 weeks were used. Before harvesting the mammary gland, the estrus stage of mice was examined by vaginal smear cytology as described previously (Byers et al., 2012; McLean et al., 2012).

### Western blotting

Cultured primary mammary cells were lysed in RIPA buffer (Beyotime) with 1 mM phenylmethanesulfonyl fluoride (200 μl per well) on ice for 30 min with a good shaking. The mixture was centrifuged at 4°C, 12000 rpm, for 15 min. The supernatant was collected in a clean tube, subjected to protein denaturation in a water bath at 98°C for 10 min, and stored at −20°C. Proteins were separated by sodium dodecylsulfate–polyacrylamide gel electrophoresis and transferred to polyvinylidene difluoride membranes (Millipore) for antibody incubation. The proteins were detected by ECL chemiluminescence kit (Monad).

### Transcriptomic analysis

DrTom network platform of BGI (https://biosys.bgi.com) was used to perform the GO and KEGG analyses. GSEA v4.1.0 was used to perform the GSEA on various characteristic gene signatures.

### Statistical analysis

All data are shown as mean ±  standard deviation (SD). Unless otherwise indicated, unpaired Student's *t*-test was used for comparisons between two samples, and *P* < 0.05 was considered statistically significant. Analyses were performed using GraphPad Prism 7 or GraphPad Prism 8 software. Three independent experiments were carried out for statistical analysis unless specified otherwise.

### Data availability

RNA-seq data generated in this study are publicly available in GEO dataset under accession number GSE243652.

## Supplementary Material

mjae020_Supplemental_File

## Notes

**Author contributions:** conception: C.C., Z.Y., and M.Z.; research design: C.C., Z.Y., Y.X., and M.Z.; cell experiments: Z.Y. and Y.X.; animal experiments: Z.Y. and Y.X.; data collection: Z.Y.; data analyses and interpretation: Z.Y.; preparation of figures: Z.Y.; manuscript writing: C.C., M.Z., and Z.Y. All authors provided suggestions and edits to the final version of the manuscript.
