# Supplementary material for: Sympathetic nerve signals: orchestrators of mammary development and stem cell vitality
Source: J Mol Cell Biol. 2024 May 13;16(5):mjae020. doi: 10.1093/jmcb/mjae020 (PMC11520406; doi:10.1093/jmcb/mjae020)
Supplement: mjae020_Supplemental_File [file mjae020_supplemental_file.pdf]

Supplementary Figure S1

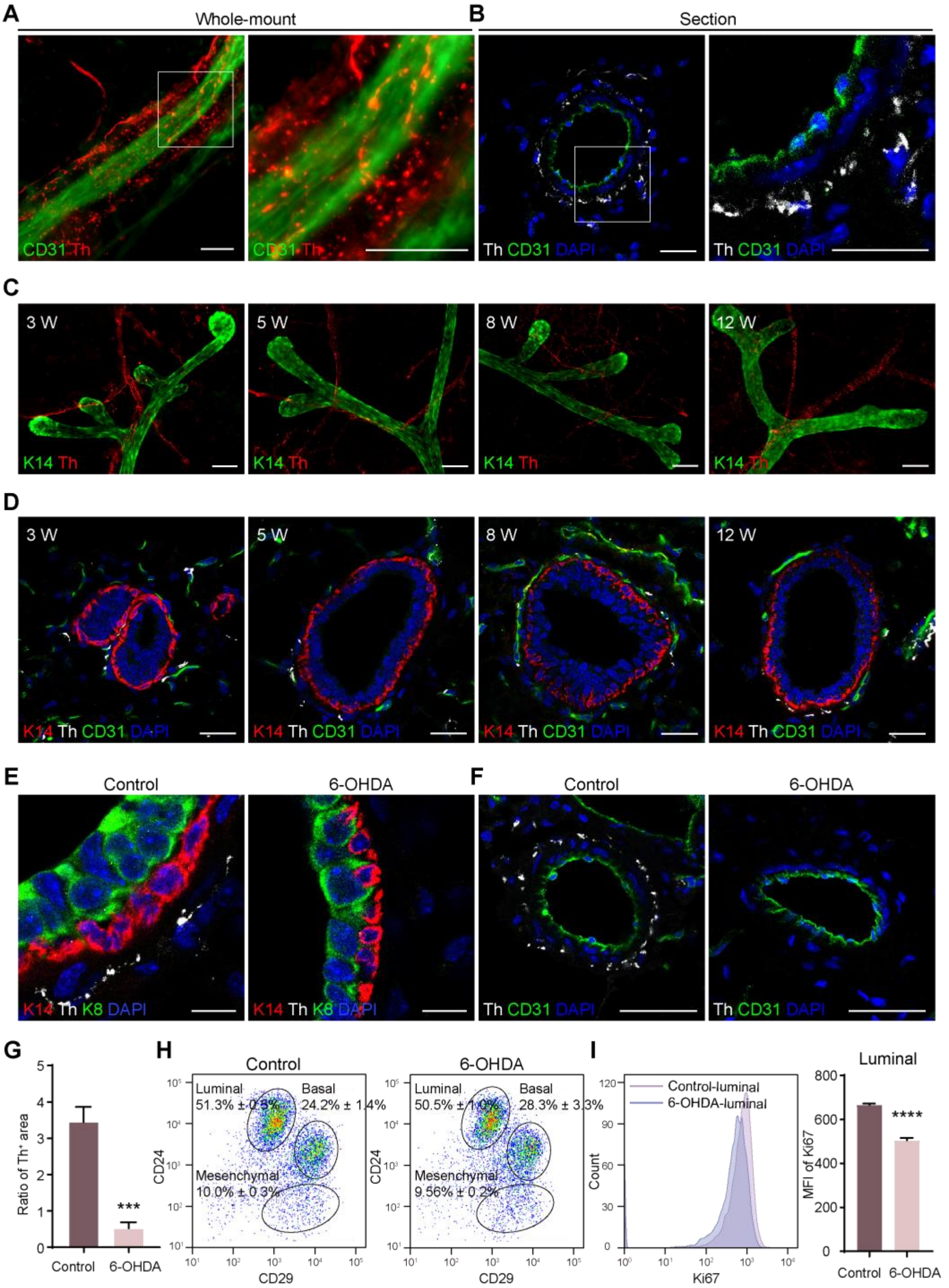

Supplementary Figure S1: Sympathetic Innervation in the Mammary Fat Pad and Its Impact on Mammary Epithelial Cell

(A) Whole-mount staining images depict the presence of Th^{+} sympathetic nerves and CD31^{+} vasculature within the mouse mammary fat pad. Scale bar: 100 µm.

(B) Immunofluorescent staining of Th and CD31 in the mouse mammary fat pad. Scale bar: 25 μm.

(C) Whole-mount staining of K14 and Th in mammary glands of 3 W, 5 W, 8 W, and 12 W female mice. Scale bars: 100 μm.

(D) Immunofluorescent staining of K14, Th and CD31 in mammary glands of 3 W, 5 W, 8 W, and 12 W female mice. Scale bars: 25 μm.

(E) Immunofluorescent staining of Th, K14 and K8 in both control and sympathectomized mouse mammary glands. Scale bar: 10 μm.

(F) Immunofluorescent staining of Th and CD31 in both control and sympathectomized mouse mammary glands indicated fewer sympathetic nerve fibers in 6-OHDA compared with the Control. Scale bar: 50 μm.

(G) Quantitative assessment of Th^+ fibers in the mammary glands of female mice treated with saline or 6-OHDA.

(H) FACS analysis conducted to identify subpopulations of mammary epithelial cells labeled by CD24 and CD29 in control and sympathectomized mammary glands.

(I) FACS analysis of Ki67 expression in luminal cells (left panel). Quantification of MFI of Ki67-PE (right panel).

The results are representative of 3 independent experiments. Data are represented as mean ± SD. Student's *t-test*: ***$p < 0.001$, ****$p < 0.0001$.

Supplementary Figure S2

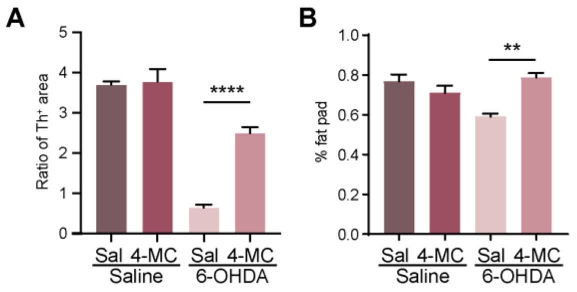

Supplementary Figure S2: Effect of Neuroprotection on the Mammary Duct Elongation

(A) Quantification of Th^{+} fibers in mammary gland of mice treated with saline, 4-MC, 6-OHDA or 4-MC + 6-OHDA.

(B) Quantification of mammary duct elongation in control, 4-MC, 6-OHDA or 4-MC + 6-OHDA female mice.

The results are representative of 3 independent experiments. Data are represented as mean ± SEM. Student's *t-test*: **$p < 0.01$, ****$p < 0.0001$.

Supplementary Figure S3

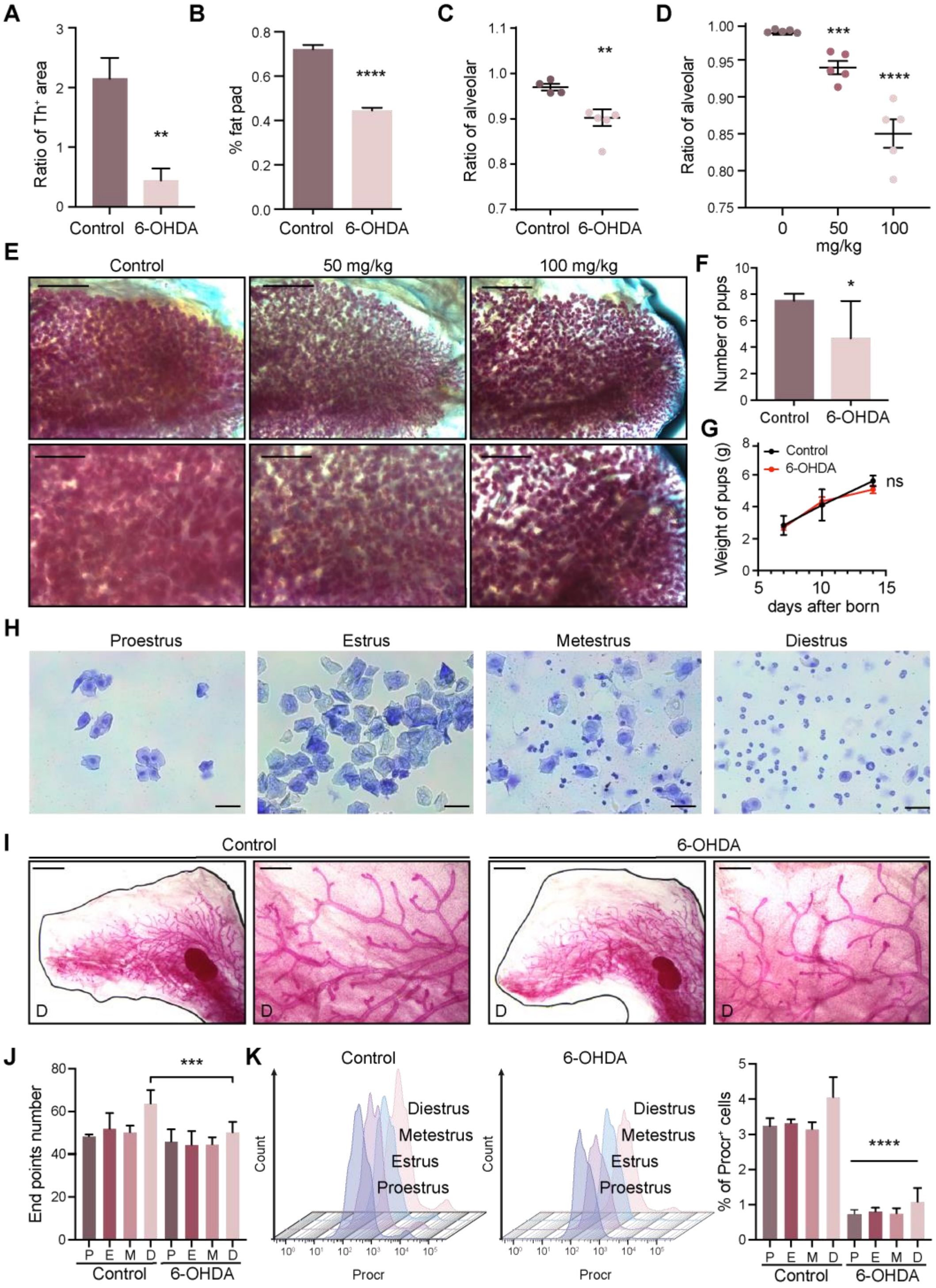

Supplementary Figure S3: Sympathetic Nerve is Essential for Alveolar Formation and Mammary Development in Estrus Cycle

(A) Quantification of Th^+ fibers in mammary gland of pregnant mice treated with saline or 6-OHDA.

(B) Quantification of mammary duct elongation in pregnant mice treated with saline or 6-OHDA.

(C) Quantification of alveolar cells in mammary gland of pregnant mice treated with saline or 6-OHDA.

(D) Quantification of alveolar cells in the mammary gland of pregnant mice treated with saline or varying concentrations of 6-OHDA.

(E) Carmine staining showcasing the impact of different concentrations of 6-OHDA on mammary alveolar formation. Scale bars: 2 mm and 1 mm.

(F) Number of pups produced by pregnant mice treated with saline or 6-OHDA.

(G) Weight changes of pups during lactation.

(H) Virginal cytology representing each stage of estrus cycle. Stages include proestrus, estrus, metestrus and diestrus. Scale bars: 50 µm.

(I) Carmine staining of mammary glands from mice treated with saline or 6-OHDA at diestrus stage. Scale bars: 2 mm and 500 µm.

(J) Quantification of end points indicated that mammary ductal side branches decreased at diestrus stage in 6-OHDA treated mice.

(K) FACS analysis of $Procr^+$ basal cells in mice at different estrus stages indicated reduced $Procr^+$ MaSCs after 6-OHDA treatment.

The results are representative of 3 independent experiments. Data are represented as mean ± SD. Student's *t-test*: ns, not significant, $^*p < 0.05$, $^{**}p < 0.01$, $^{***}p < 0.001$, $^{****}p < 0.0001$.

Supplementary Figure S4

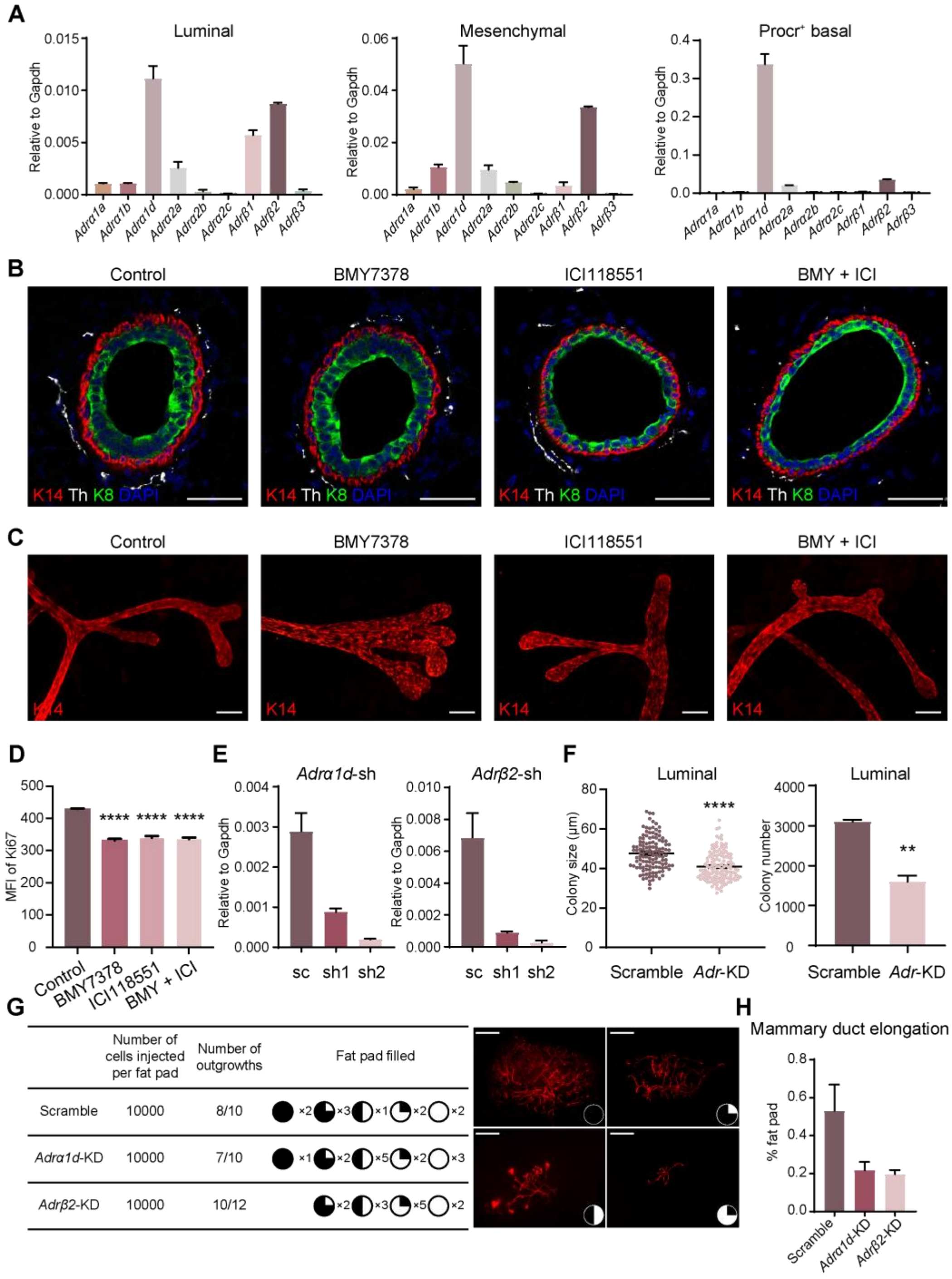

Supplementary Figure S4: Mammary Development and Expansion of MaSCs Mediated by Adrenergic Receptors

(A) RT-qPCR analysis of the expression of adrenergic receptors in mammary epithelial cell populations: luminal, mesenchymal and Procr$^+$ basal cells.

(B) Immunofluorescent staining of Th, K14 and K8 in mammary gland from female mice treated with saline or antagonists. Scale bar: 50 µm.

(C) Whole-mount K14 staining of the mammary duct from female mice treated with saline or antagonists. Scale bar: 100 μm.

(D) Quantification of MFI of Ki67-PE in basal cells of female mice treated with saline or antagonists.

(E) RT-qPCR analysis of *Adrα1d* and *Adrβ2* knockdown efficiency.

(F) Colony size (left panel) and colony number (right panel) of *Adr*-KD and control luminal cells in 3D culture.

(G) The numbers and size (shown as the percentage of fat pad filled right panel) of mammary outgrowths in transplantation.

(H) Quantitative analysis of mammary duct elongation of outgrowths in transplantation.

The results are representative of 3 independent experiments. Data are represented as mean ± SEM. Student's *t-test*: $^{**}p < 0.01$, $^{****}p < 0.0001$.

Supplementary Figure S5

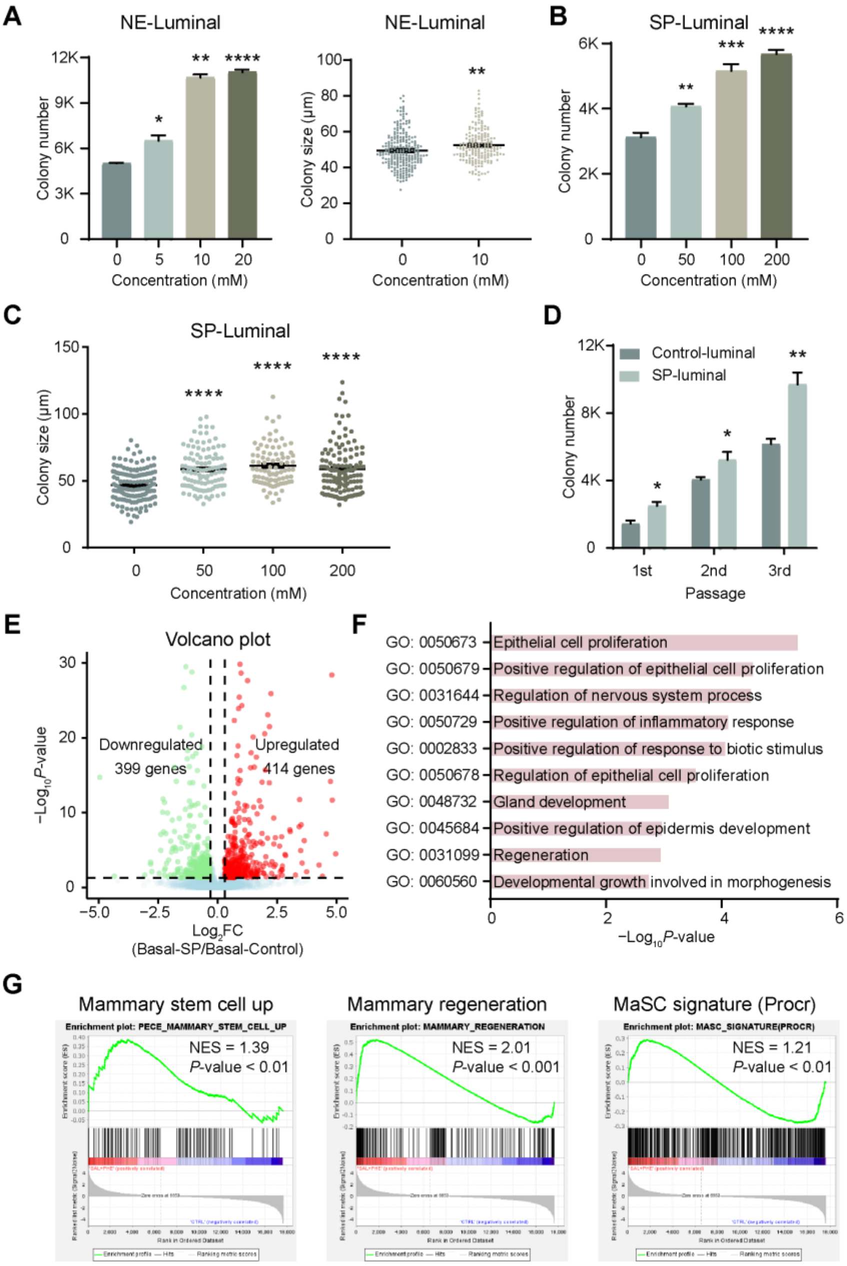

Supplementary Figure S5: The Effect of Neural Signals Activation on Basal and Luminal Colonies

(A) Colony number (left) and colony size (right) of luminal cells treated with different concentrations of NE.

(B) Colony number of luminal cells treated with SP at gradient concentration in 3D culture.

(C) Colony size of luminal cells treated with SP at gradient concentration in 3D culture.

(D) The number of luminal cell colonies treated with SP in serial passages.

(E) A volcano plot illustrating the differentially expressed genes in colonies formed by control and SP-treated basal cells.

(F) GO enrichment analysis of up-regulated differentially expressed genes in colonies formed by SP-treated basal cells.

(G) GSEA demonstrating that the gene signatures related to "Mammary stem cell up", "Mammary regeneration", and "MaSC (Procr-labeled)" were up-regulated in SP-treated basal colonies compared to control basal colonies.

The results are representative of 3 independent experiments. Data are represented as mean ± SD. Student's *t-test*: *$p < 0.05$, **$p < 0.01$, ***$p < 0.001$, ****$p < 0.0001$.

Supplementary Table S1: Primers for lentiviral shRNA constructs

shRNA	Primers
Scramble-shRNA	5'-TCCTAAGGTTAAGTCGCCCTCG-3'
Adrα1d-shRNA-1	5'-CTCTTCCGTATGCTCCTTCTA-3'
Adrα1d-shRNA-2	5'-GAAGCAGTGTCCCTAAATGTT-3'
Adrβ2-shRNA-1	5'-CCTCATCCCTAAGGAAGTTTA-3'
Adrβ2-shRNA-2	5'-CGGCTACTCTAGCAATAGCAA-3'

Supplementary Table S2: Primers for RT-qPCR

Gene	Primers
Gapdh-F	5'-AGGTCGGTGTGAACGGATTTG-3'
Gapdh-R	5'-TGTAGACCATGTAGTTGAGGTCA-3'
Adrα1a-F	5'-CTAAGGCCATTCTACTTGGGGT-3'
Adrα1a-R	5'-CGAGTGCAGATGCCGATGA-3'
Adrα1b-F	5'-CGGACGCCAACCAACTACTT-3'
Adrα1b-R	5'-AACACAGGACATCAACCGCTG-3'
Adrα1d-F	5'-AGTGGGTGTCTTCCTAGCC-3'
Adrα1d-R	5'-GCCTAGAACCTCCATAGTGGC-3'
Adrα2a-F	5'-GTGACACTGACGCTGGTTTG-3'
Adrα2a-R	5'-CCAGTAACCCATAACCTCGTTG-3'
Adrα2b-F	5'-TCTTCACCATTTTCGGCAATGC-3'
Adrα2b-R	5'-AGAGTAGCCACTAGGATGTCG-3'
Adrα2c-F	5'-CTGTGGTGGGTTTCCTCATCG-3'
Adrα2c-R	5'-ACTTGCCCGAAGTACCAGTAG-3'
Adrβ1-F	5'-CTCATCGTGGTGGGTAACGTG-3'
Adrβ1-R	5'-ACACACAGCACATCTACCGAA-3'
Adrβ2-F	5'-GGGAACGACAGCGACTTCTT-3'
Adrβ2-R	5'-GCCAGGACGATAACCGACAT-3'
Adrβ3-F	5'-AGAAACGGCTCTCTGGCTTTG-3'

Adrβ3-R	5'-TGGTTATGGTCTGTAGTCTCGG-3'

Supplementary Table S3: Antibodies for immunofluorescence and whole-mount staining

Antibodies	Source
Rabbit anti-Krt14	BioLegend
Rat anti-Krt8	DSHB
Rabbit anti-tyrosine hydroxylase	Millipore
Rat anti-FITC-conjugated CD31	BD Biosciences
Guinea pig anti-Krt14	Wuhan Dia-an Biotechnology Company
Rabbit anti-Ki67	Cell Signaling Technology
Rabbit anti-milk	Nordic Immunological Laboratories

Supplementary Table S4: Antibodies for western blot

Antibodies	Source
β-actin	Proteintech
MEK1/2	Selleck
phospho MEK1/2	ABclonal
ERK1/2	Sabbiotech
phospho ERK1/2	Cell Signaling Technology
